# Has COVID-19 changed the stock return-oil price predictability pattern?

**DOI:** 10.1186/s40854-021-00277-7

**Published:** 2021-08-16

**Authors:** Fan Zhang, Paresh Kumar Narayan, Neluka Devpura

**Affiliations:** 1 School of Public Finance and Taxation, Zhejiang University of Finance and Economics, Hangzhou, China; 2 Monash Business School, Monash University, Melbourne, Australia; 3 Department of Statistics, Faculty of Applied Sciences, University of Sri Jayewardenepura, Nugegoda, Sri Lanka

**Keywords:** COVID-19, Oil prices, Stock returns, E31, E37, F37

## Abstract

In this paper, we examine if COVID-19 has impacted the relationship between oil prices and stock returns predictions using daily Japanese stock market data from 01/04/2020 to 03/17/2021. We make a novel contribution to the literature by testing whether the COVID-19 pandemic has changed this predictability relationship. Employing an empirical model that controls for seasonal effects, return-related control variables, heteroskedasticity, persistency, and endogeneity, we demonstrate that the influence of oil prices on stock returns declined by around 89.5% due to COVID-19. This implies that when COVID-19 reduced economic activity and destabilized financial markets, the influence of oil prices on stock returns declined. This finding could have implications for trading strategies that rely on oil prices.

## Introduction

We aim to provide preliminary evidence on the role of COVID-19 in influencing the stock returns-oil price predictability relationship. The effect of oil price on stock returns has been studied extensively, with over 100 papers published since 2000; for a survey of this literature, see Smyth and Narayan (2018). The relationship between oil prices and stock returns is inspired by the cash flow hypothesis (see Fisher 1930; Williams 1938), which perceives a positive and a negative effect of oil price on stock returns. The negative effect emerges as higher oil prices reduce cash flows, earnings, and dividends by increasing production costs. Stock returns are negatively impacted as a result of declining dividends. The positive effect emerges when oil prices decline during a phase such as the COVID-19 pandemic, which saw oil prices subdued and at historical lows (Devpura and Narayan 2020). Such low oil prices have a positive effect on cash flows because they reduce the cost of production, keeping other factors constant. The positive and negative effect of oil prices on stock returns are also a result of the response of monetary authorities to changes in oil prices. If changes in oil prices result in higher inflation and the objective is to control inflation, the central bank will raise the short-term interest rate, and vice versa. With a higher interest rate, excess stock returns will decline. The effect is the opposite when inflation is low, and the monetary objective is to control inflation.

Aside from that discussed above, we hypothesize that COVID-19 has influenced the strength of the stock returns-oil price relationship. This is motivated by the recent empirical evidence suggesting that the pandemic has significantly influenced the energy markets (Fu and Shen 2020; Gil-Alana and Claudio-Quiroga 2020), cryptocurrencies (see Conlon and McGee 2020; Corbet et al. 2020; and Grobys 2020) and the financial and economic systems (Gu et al. 2020; Haroon and Rizvi 2020a,b; Iyke 2020a, b) globally. How would stock returns react to oil prices? This is easy to discern. At the outset, the theory that motivates the reaction of stock returns to oil prices should be examined. This relationship has been tested in the literature based on the underreaction theory (see Hong and Stein 1999; Hong et al. 2007), which in turn is built on the premise that a change in oil prices results in a gradual diffusion of information. The central theme of the gradual diffusion hypothesis is that investors tend to underreact to oil price changes (as in our case) when such changes induce a significant effect on economic activity, due to which investors are less certain of understanding the impact of the shock. The pandemic has created a scenario where investors are unable to predict the effect of the decline in oil prices on their investment decisions. As a result, we hypothesize that investors are likely to underreact and adopt a conservative approach to investment decisions due to COVID-19. We, therefore, propose that the influence of oil price on stock returns is likely to become weaker than normal (non-COVID-19) times.

We focus on the Japanese stock market, based on the recent COVID-19 literature on Japan. We refer to Narayan et al. (2020), who have eloquently discussed the motivations behind this choice. They argue that the Japanese market is unique given the country’s COVID-19 situation and the reactions of both the government and citizens. Japan stands out in this pandemic vis-à-vis other developed countries because: (a) it implemented a travel ban policy much faster; (b) departing from the G7’s response to COVID-19, it avoided locking down the country; and (c) recorded deaths in Japan have been significantly lower than in other G7 countries, such as the USA, the UK, and Italy. In other words, as Narayan et al. (2020) argue, Japan is a model of success in managing and containing the virus.

The above literature suggests modeling the effects of COVID-19 using a time-varying approach. The need to treat the effects of COVID-19 in a time-varying manner is evident: the pandemic represents the largest and most disturbing shock to the global economic system (Sha and Sharma 2020; Sharma 2020; Tisdell 2020). The pandemic, therefore, represents the largest structural shift in any statistical and economic relationship regardless of theory. The pandemic began over a year ago; significant time has elapsed, and the effects of the pandemic have persisted. This points to possible time-varying effects.

Our aim of testing the predictability of the oil price-stock returns relationship is novel when compared to related recent studies such as those of Prabheesh et al. (2020) and Salisu et al. (2020a, b) because we examine the change in relationship during the pandemic period rather than during the pre-pandemic period. We contribute to the literature by specifically showing that this well-known relationship substantially weakened due to COVID-19. The popular empirical framework employed in the literature is based on time-series regression models and/or predictability models. While we test the same traditional hypothesis using the same basic regression framework, we improve on the modeling by (a) controlling for heteroskedasticity (since we employ daily data); (b) accounting for the endogeneity of oil prices; and (c) controlling for the persistency of oil prices. We perform (a)–(c) by applying the Westerlund and Narayan (2015) approach.^1^ In addition, we control for seasonality. Our research question and approach allows us to demonstrate that while the ability of oil prices to predict stock returns is retained even when a pandemic has an economic effect as devastating as COVID-19, the relationship is substantially weaker as a result of the pandemic. Our finding implies that the relevance of oil prices to stock returns survives (but weakens) even during an unprecedented crisis.

Our second contribution is to the COVID-19 literature, which has accumulated a rich body of findings demonstrating the pandemic’s impact on the functioning of various financial and economic systems globally and the emergence of policy challenges in attempts to mitigate its effects (see Padhan and Prabheesh 2021). We add to this literature on oil price-stock returns during the pandemic (see Prabheesh et al. 2020; Salisu et al. 2020a, b) by demonstrating the relevance of oil prices in influencing stock returns during a pandemic. Our empirical investigation finds that the effects of oil prices on stock returns declined by around 89.5% due to COVID-19. This evidence is robust to different empirical specifications, use of alternative estimators, different sample periods, and controls for heteroskedasticity, persistency, and endogeneity.

## Data and results

### Preliminary observations from the data

This section presents our dataset. A summary of all variables used is presented in Table 1; see column 1 and table notes. A descriptive statistic on those variables follows. The frequency of data is daily and covers the sample from 01/04/2010 to 03/17/2021. This sample presents us with 2923 daily observations. Consistent with our hypothesis that the stock returns-oil price relationship was influenced by COVID-19, we split the data sample into three sub-samples, which are explained in sub-section B.

**Table 1:** Descriptive statistics

Variable	Mean	AR(1)	Maximum	Minimum	SD	Skewness	NP test	Prob of JB	No. of Obs
Panel A: full sample 01/04/2010 to 03/17/2021									
* R*	0.04	− 0.04	7.73	− 11.15	1.3	− 0.46	− 1.01***	0	2923
* RV*	22.64	0.96	69.88	12.19	6.57	1.68	− 0.03***	0	2923
* OIL($)*	69.21	0.99	113.93	− 37.63	23.1	0.07	− 0.01	0	2923
* GOP*	− 0.09	0.28	35.02	− 305.97	6.68	− 34.84	− 0.79***	0	2923
Panel B: pre-COVID-19 sample 1, 01/04/2010 to 12/30/2019									
* R*	0.03	− 0.05	7.43	− 11.15	1.27	− 0.59	− 1.09***	0	2606
* RV*	22.24	0.96	69.88	12.19	6.07	1.42	− 0.05***	0	2606
* OIL($)*	72.45	1	113.93	26.21	21.95	0.03	− 0.01	0	2606
* GOP*	0.01	− 0.06	14.68	− 10.17	2.08	0.27	− 1.05***	0	2606
Panel C: COVID-19 Sample 12/31/2019 to 03/17/2021									
* R*	0.07	0.05	7.73	− 6.27	1.51	0.13	− 0.98***	0	317
* RV*	25.9	0.97	60.67	13.2	9.14	1.69	− 0.07	0	317
* OIL($)*	42.55	0.94	66.09	− 37.63	12.76	− 1.01	− 0.05	0	317
* GOP*	− 0.96	0.31	35.02	− 305.97	19.41	− 13.1	− 0.8***	0	317
Panel D: pre-COVID-19 sample 2, 10/01/2018 to 12/30//2019									
* R*	− 0.01	− 0.01	3.81	− 5.14	1.03	− 0.66	− 0.81***	0	326
* RV*	18.42	0.97	32.25	12.98	3.8	1.2	− 0.12	0	326
* OIL($)*	57.34	0.96	76.41	44.41	5.65	0.75	− 0.04	0	326
* GOP*	− 0.03	− 0.07	14.68	− 7.9	2.21	0.32	− 1.06***	0	326

This table reports descriptive statistics mean value, the first-order autoregressive (AR(1)) coefficient, maximum value, minimum value, standard deviation (Std. Dev.), skewness, The Narayan and Popp (NP, 2010) structural break unit root test results, the Jarque–Bera (JB) test which examines the null hypothesis of normality (we report its *p* value), and finally the number of observations in each sample (No. of Obs.). Panel A presents the results for the full sample period (01/04/2010 to 03/17/2021), Panel B contains results for the pre-COVID-19 sample 1 (01/04/2010 to 12/30/2019), Panel C reports the descriptive statistics for the COVID-19 sample from 12/31/2019 to 03/17/2021, and Panel D contains results for the pre-COVID subsample 2, from 10/01/2018 to 30/12/2019. The variables are *R* is the log percentage return of the Nikkei price index; $$RV$$ is the Nikkei stock average volatility index, Crude Oil-WTI spot price (*OIL($)*), and finally *GOP* is the growth rate in the WTI spot price of oil. Lastly, *** denotes statistical significance at the 1% level

As sub-section B shows by way of an empirical model, our dependent variable is the Japanese stock price returns (which is the log percentage returns of the Nikkei price index, *R*). We use a proxy for Japanese stock market volatility. For this purpose, we use the Nikkei stock average volatility index (*RV*), which captures the expected degree of future fluctuation in *R*, with a higher value reflecting large fluctuations to be expected in the future. Our main predictor variable is the growth rate in oil prices. We proxy oil prices with the West Texas Intermediate (WTI) crude oil spot price (*GOP*). All data are obtained from Datastream.

**Fig. 1:**
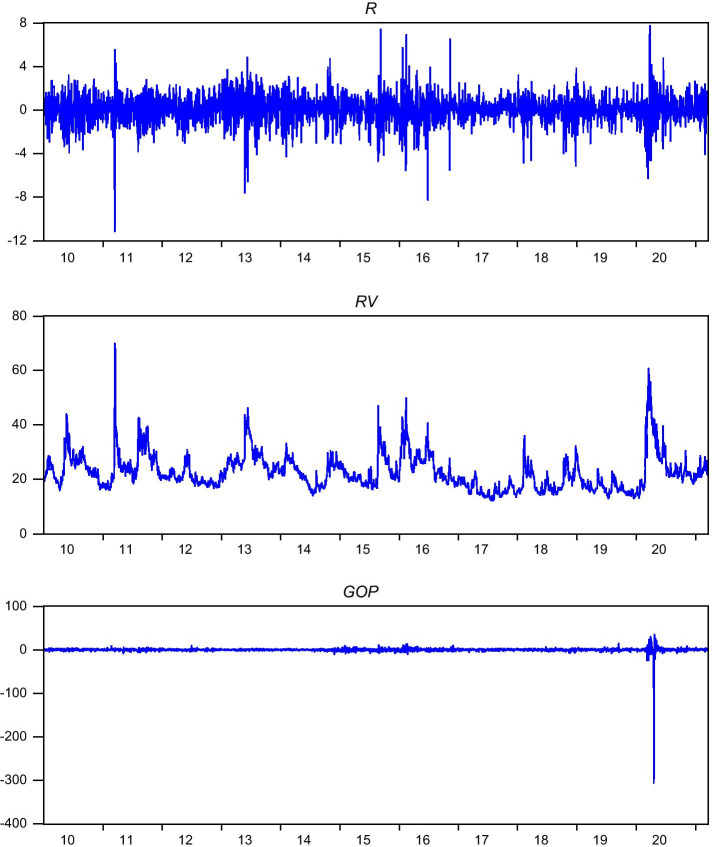
Full sample illustrations. The figure illustrates the line charts for variables namely, *R* is the log percentage return of the Nikkei price index; $RV$ is the Nikkei stock average volatility index, finally *GOP* is the growth rate in the WTI spot price of oil

Figure 1 plots the key data series—namely, *R*, *RV*, and *GOP*. Figure 1 indicates that all variables appear disturbed during the onset of COVID-19. The data indicates a clear pattern during the COVID-19 period that distinguishes it from the pre-COVID-19 period. We explore this feature of the data from the perspective of the oil price—stock return predictability relationship.

### Results

We test for predictability relationship—the ability of oil prices to predict Japanese stock returns—using a daily predictability model motivated by Garcia (2013). We use a general predictability model, as follows:

1 $$R_{t} = \alpha + \beta_{1} GOP_{t - 1} + \beta_{2} R_{t - 1} + \beta_{3} RV_{t - 1} + \beta_{4} MON_{t} + \beta_{5} TUE_{t} + \beta_{6} THU_{t} + \beta_{7} FRI_{t} + \varepsilon_{t}$$

where ${R}_{t}$ is the Japanese stock market returns which we proxy using the log percentage return of the Nikkei price index; $RV$ is the Nikkei stock average volatility index; and *MON*, *TUE*, *THU*, and *FRI* are the day of the week (Monday, Tuesday, Thursday, and Friday) dummy variables. The Wednesday dummy is excluded to avoid the dummy variable trap. The one period lagged stock returns are included to control for return persistency, and the *RV* is used to control for market volatility. The model is estimated using ordinary least squares regression (OLS) and standard errors are corrected using Newey and West (1987). We consider a maximum of 8 lags (where the optimal lag length is chosen using the Schwarz information criterion) to accommodate heteroskedasticity and autocorrelation. We also estimate Eq. (1) using the Westerlund and Narayan (2015) flexible generalized least squares estimator, which controls for any heteroskedasticity, persistency, and endogeneity in the model and variables.

We also control for seasonality effects by proposing the following regression model:

2 $$\begin{aligned} R_{t} & = \alpha + \beta_{1} GOP_{t - 1} + \beta_{2} R_{t - 1} + \beta_{3} RV_{t - 1} + \beta_{4} MON_{t} + \beta_{5} TUE_{t} + \beta_{6} THU_{t} + \beta_{7} FRI_{t} \\ & \quad + \beta_{8} JAN_{t} + \beta_{9} JAN_{t} + \beta_{10} FEB_{t} + \beta_{11} MAR_{t} + \beta_{12} APR_{t} + \beta_{13} MAY_{t} + \beta_{14} JUN_{t} \\ & \quad + \beta_{15} AUG_{t} + \beta_{16} SEP_{t} + \beta_{17} OCT_{t} + \beta_{18} NOV_{t} + \beta_{19} DEC_{t} + \varepsilon_{t} \\ \end{aligned}$$

In this regression, all variables are as previously defined except we now augment Eq. (1) with monthly dummy variables, namely *JAN, FEB, MAR, APR, MAY, JUN, AUG, SEP, OCT, NOV,* and *DEC* (where the month of July is dropped due to the dummy trap issue) to capture the seasonal effects. The models are estimated as described with respect to Eq. (1).

The descriptive statistics of the data are reported in Table 1. We consider four sample periods to draw conclusions on the effect of COVID-19. The first sample is the full sample covering 01/04/2010 to 03/17/2021 (2923 observations). The second period is what we refer to as the pre-COVID-19 sample: 01/04/2010 to 12/30/2019 (2606 observations). The third sample is the current COVID-19 sample (12/31/2019 to 03/17/2021 = 317 observations). For the last sample period, we match this current COVID-19 sample with a pre-COVID-19 sample covering roughly the same number of observations by taking a sub-sample period of 10/01/2018 to 12/30/2019 (326 observations). The last two sub-samples allow for a direct comparison and act as a robustness testing sample.

The most interesting statistic is with regard to the mean returns in the COVID-19 period compared to the pre-COVID sub-samples. In the COVID-19 period, mean returns have a daily average of 0.07%. Over the same period a year ago, average daily returns are 0.01% (panel D). In a much lengthier sample covering pre-COVID-19 data, average daily returns are 0.03% (Panel B). Similarly, we notice that the volatility of the Japanese stock market has doubled in the COVID-19 period compared to the pre-COVID-19 period.

The main implication of these descriptive statistics is that the Japanese stock market behaved very differently during the COVID-19 phases compared to the pre-COVID-19 phase. This is consistent with the observation made from examining Fig. 1. The question arises: Is this pattern of behavior also reflected in the predictability of Japanese stock returns when the oil price is the information variable? The results that address this question are presented in Table 2. Two sets of results are reported: One model is estimated with no control variables—that is, $\beta_{2}$ to $\beta_{7}$ is set to zero, while in the second model all control variables are included, as in Eq. (1). Two estimators are used: Panel A presents results based on the heteroskedasticity and autocorrelation consistent with OLS estimates while Panel B presents the corresponding estimates obtained using the WN flexible generalized least squares. The results are robust with regard to the accounting of control variables and estimators. Since Eq. (1) is our main model, we discuss results only from the full-scale model. We give more weight to the WN estimator because apart from controlling for heteroskedasticity it obviates any issues with respect to variable persistency and endogeneity (Westerlund and Narayan 2012).

**Table 2:** The effect of oil prices on Japanese stock returns

	Panel A: OLS estimator			Panel B: WN-FGLS estimator				
Sample periods	Model with no controls	Model 1	***R***^2^ (%)	Model with no controls	Model 1	***R***^2^ (%)	Model 2 (seasonal)	***R***^2^ (%)
Full-sample	0.0161** (1.9865)	0.0168** (2.0599)	0.77	0.0201** (1.9911)	0.0206**(2.0404)	0.80	0.0210** (2.0884)	0.84
COVID-19 sample	0.0072*** (6.8010)	0.0086*** (6.6904)	1.96	0.0106*** (4.6034)	0.0126*** (5.5035)	2.14	0.0138*** (4.7074)	1.28
Pre-COVID-19 Sample 1	0.1114*** (6.7405)	0.1137*** (6.8842)	3.63	0.1664*** (7.9654)	0.1691*** (7.8464)	4.31	0.1696*** (7.8641)	4.36
Pre-COVID-19 Sample 2	0.0453** (2.0306)	0.0533** (2.2773)	0.47	0.1260*** (3.6939)	0.1339*** (3.6983)	2.74	0.1317*** (3.4613)	2.76

This tables reports predictability test results based on the following time-series regression (Model 1):

$$R_{t} = \alpha + \beta_{1} GOP_{t - 1} + \beta_{2} R_{t - 1} + \beta_{3} RV_{t - 1} + \beta_{4} MON_{t} + \beta_{5} TUE_{t} + \beta_{6} THU_{t} + \beta_{7} FRI_{t} + \varepsilon_{t}$$

The second model which we refer to as Model 2 is of the form:

$$R_{t}  = \alpha + \beta_{1} GOP_{t - 1} + \beta_{2} R_{t - 1} + \beta_{3} RV_{t - 1} + \beta_{4} MON_{t} + \beta_{5} TUE_{t} + \beta_{6} THU_{t}  + \beta_{7} FRI_{t} + \beta_{8} JAN_{t} + \beta_{9} JAN_{t} + \beta_{10} FEB_{t} + \beta_{11} MAR_{t} + \beta_{12} APR_{t} + \beta_{13} MAY_{t}  + \beta_{14} JUN_{t} + \beta_{15} AUG_{t} + \beta_{16} SEP_{t} + \beta_{17} OCT_{t} + \beta_{18} NOV_{t} + \beta_{19} DEC_{t} + \varepsilon_{t} $$

where $R_{t}$ is the Japanese stock market returns (log percentage returns of the Nikkei price index); *GOP* is the growth rate in the WTI spot price of oil; $RV$ is the Nikkei stock average volatility index; *MON*, *TUE*, *THU*, and *FRI* are the day-of-the-week (Monday, Tuesday, Thursday and Friday) dummy variables and *JAN, FEB, MAR, APR, MAY, JUN, AUG, SEP, OCT, NOV* and *DEC* are to capture the seasonal effects respectively. The models are estimated using OLS with standard errors corrected using the Newey and West (1987) procedure such that the estimates are autocorrelation and heteroskedasticity consistent (Panel A). We also estimate the model using the Westerlund and Narayan (2015) flexible generalized least squares (WN-FGLS) estimator which makes the estimates heteroskedasticity, persistency and endogeneity consistent (Panel B). We only report the main slope coefficient relating to $\beta_{1}$ = 0 that examines the null hypothesis that *GOP* does not predict stock returns. Four sample periods are considered: the full sample period covers 01/04/2010 to 03/17/2021; the COVID-19 sample has data for the 12/31/2019 to 03/17/2021 period; the pre-COVID-19 Sample 1 covers the 01/04/2010 to 12/30/2019 period; and the pre-COVID-19 Sample 2 has data for the 10/01/2018 to 12/30/2019 period. Lastly, ** (***) denote statistical significance at the 5% (1%) level.

We present the results of the OLS estimator as reported in Panel A of Table 2. Over the full sample period and the three sub-sample periods, in a model without any controls, the oil price positively predicts stock price returns. We observe a considerable difference in the effect of oil prices on stock price returns over the pre-COVID-19 period compared to the COVID-19 period. Overall, the effect is much smaller during the COVID-19 period. This trend in results holds when the regression model is augmented with control variables, suggesting that even though the OLS model does not remedy issues of heteroskedasticity, persistency, and endogeneity of the variables (statistical concerns), there is clear evidence of the oil price-stock returns relationship weakening in the COVID-19 period. Is this evidence robust to modeling (a) those statistical concerns and (b) seasonality in the data? The answer to this question is presented in Panel B of Table 2. Three results are pertinent. First, we see that in the model without controls, oil prices once again predict stock price returns regardless of the data sample, and the relationship between the two variables weakens substantially during the COVID-19 period (0.0106, *t*-statistic = 4.60) compared to the pre-COVID-19 period (0.1691, *t*-statistic = 7.97). Second, this pattern of the oil price influencing stock returns is repeated in Model 1 which includes control variables, suggesting that the results are robust.

Third, these findings allow us to go a step further and ascertain whether the results are also robust to seasonality in the data. The results are presented as Model 2 in Table 2. Over the full sample period (that includes the COVID-19 sample), oil prices predict stock returns positively (0.021, *t*-statistic = 2.08). This predictability is stronger when we exclude the COVID-19 sample. Oil prices predict stock returns to a larger extent in this period: 0.17 (*t*-statistic = 7.89). The strength of this predictability declines to 0.014% (*t*-statistic = 4.71) in the COVID-19 sample. We note that our pre-COVID-19 sample has 2606 observations while the COVID-19 sample has a small fraction of this sample (only 317 observations). We therefore test the robustness of our finding that the ability of oil prices to predict Japanese stock returns weakened in the COVID-19 period by ensuring that the result we obtain is not sensitive to the choice of a large sample period. To do so, we choose another sample that corresponds to the size of the COVID-19 sample covering an almost equivalent period a year ago. The predictive slope coefficient is 0.13 (*t*-statistic = 3.46), implying that the ability of oil prices to predict stock returns declined in the COVID-19 period regardless of how we measure the pre-COVID-19 sample. Overall, we observe that the strength of the oil price-stock returns relationship declined in the COVID-19 period by around 89.5% compared to the pre-COVID-19 period.

The reason for this loss in the strength of the relationship is fairly evident. First, COVID-19 resulted in a slowdown in global economic activity. Since oil demand/supply and production/consumption are clearly dependent on economic activity, any slowdown in economic activity will result in a reduction in the production and consumption of oil. In other words, a slowdown in economic activity will negatively impact the demand and supply of oil. This will not only make the oil market (price) more volatile but will also result in a decline in oil prices. This is precisely what we observe from the raw data, as presented earlier in Sect. 2. Similarly, the stock market performance is positively related to economic activity, implying that when economic activity declines, so does the stock market. If economic activity and oil prices are both in decline, and economic activity is linked to both oil and financial markets, a reduction in oil prices will dampen stock market performance.

## Concluding remarks

Many studies indicate that oil prices predict stock returns. Faced with the current COVID-19 pandemic which has disrupted both stock and oil markets, we examine whether the predictability of the relationship between stock returns and oil prices has also been disturbed. Using daily data for Japan, we demonstrate that while oil prices predict stock returns in both the pre-COVID-19 and COVID-19 data, the predictive effect of oil prices has declined by around 89.5% in the COVID-19 compared to the pre-COVID-19 period. The main implication of our results has roots in this literature which shows that investors can devise successful trading strategies by using information on oil prices. Since the effect of oil price on stock returns has substantially weakened due to the COVID-19 pandemic, two concerns are of interest to investors. First, is the available information on oil prices during the COVID-19 pandemic still sufficient for them to devise successful trading strategies? Second, even if the answer to the first concern is affirmative, the robustness of trading strategies needs to be evaluated in light of the pandemic. We leave these questions for future research.

Our results are robust to different model specifications, sub-sample analysis, controls for seasonality and other return-related control variables, and to the accounting of data issues such as endogeneity, persistency, and heteroskedasticity. Future research can build on our findings by exploring specific factors responsible for the decline in the relationship between oil prices and stock returns. We have provided a preliminary explanation for why the relationship has weakened during the COVID-19 pandemic. Future studies can test the validity of our hypothesis by using data as they become available. In addition, we believe that future studies can consider our hypothesis in the context of oil importer and oil exporter countries and investigate whether our claim holds for these two different groups of countries. Our hypothesis can also be tested at the firm level, including for different portfolios of firms. Overall, the potential for future research on the theme we introduce remains active.

## Data Availability

All data used in this paper are downloaded from Datastream. Given our institutional subscription legal clauses data downloaded from Datastream cannot be shared publicly. However this data can be easily downloaded from Datastream by anyone who has institutional subscription.

## Abbreviations

R: Nikkei price index
RV: Nikkei stock average volatility index
WTI: West Texas Intermediate
GOP: Crude oil spot price
WN: Westerlund and Narayan

## Footnotes

1 This model has been widely used in the predictability literature; see, for instance, Salisu and Sakiru (2020) on predicting returns; Sharma (2019) on predicting inflation; and Salisu et al. (2020a, b) and Fianto et al. (2020) on predicting exchange rates.
